# The effect of surgical field suction flow rate and venous reservoir levels on gaseous microemboli transmission⋆

**DOI:** 10.1051/ject/2025067

**Published:** 2026-03-13

**Authors:** Thomas Rath, Marisa Sugden, Edward Evans, Kyle Dana, Mitchell Rentschler, Charlotte Bolch, Nathaniel H. Darban

**Affiliations:** 1 College of Health Sciences, Midwestern University 19555 N. 59th Avenue Glendale AZ 85308 USA; 2 College of Graduate Studies, Midwestern University 19555 N. 59th Avenue Glendale AZ 85308 USA; 3 Cardiovascular Perfusion Program, Lipscomb University, 1 University Park Dr Nashville TN 37204 USA; 4 Arizona College of Osteopathic Medicine, Midwestern University 19555 N. 59th Avenue Glendale AZ 85308 USA; 5 Office of Research & Sponsored Programs, Midwestern University 19555 N. 59th Avenue Glendale AZ 85308 USA

**Keywords:** Cardiopulmonary bypass, Gaseous microemboli, Perfusion, Venous reservoir, Cardiotomy suction

## Abstract

*Background*: Minimizing Gaseous microemboli (GME) introduced into the CPB circuit can help alleviate neurologic injury. This study focuses on understanding how suction flow rate and the reservoir level can influence the introduction of GME past the venous reservoir during CPB. *Methods*: An in vitro mock CPB loop filled with bovine blood was used to simulate adult CPB. A Gampt BCC-300 bubble detector measured bubble size, volume, and count at three locations: post-reservoir (venous), post-oxygenator/arterial filter (arterial), and the venous inlet to the reservoir (recirculation). Room air was added into the suction line at 200 mL/min and mixed with blood to simulate aerated suction return. Bubble transmission was measured for three minutes at three reservoir levels, 200 mL, 500 mL, and 1000 mL, and at four pump sucker flow rates: 25 RPM (0.32 L/min), 50 RPM (0.65 L/min), 75 RPM (0.99 L/min), and 100 RPM (1.32 L/min). GME count data were pooled from three commonly used, coated, disposable reservoirs/oxygenator combinations: Medtronic Affinity Fusion, Terumo CAPIOX FX25, and Sorin Inspire 8F. *Results:* A total of 284 measurements were conducted, and the data from all reservoir manufacturers were analyzed and averaged. A statistically significant interaction was noticed between roller pump suction rate and reservoir level (*p*-value < 0.0001) at the venous sensor. As the suction flow rate increased, the reservoir level decreased, or a combination of the two occurred, a significant increase in GME count was observed at the post-reservoir sensor. Analysis of the GME count from the post-oxygenator/filter sensor revealed a significant increase as the suction flow rate increased from 25 RPM to 100 RPM. *Conclusion:* A minimum effective suction flow rate and maximum practical reservoir level are recommended to prevent the transmission of GME through the cardiopulmonary bypass circuit and potentially to the patient. Care should be taken to continuously monitor these variables throughout the case and adjust them accordingly.

## Introduction

The presence of iatrogenic gaseous micro-emboli (GME) introduced into the circulatory system during cardiac surgery is ubiquitous and well-documented in the literature. GME is defined as air that the naked eye cannot see. In vivo clinical studies and in vitro benchtop work demonstrate that “air is there” and that we can do better for our patients.

Clinical (in vivo) studies utilizing transcranial doppler (TCD) ultrasonography of the middle cerebral artery of patients undergoing cardiopulmonary bypass (CPB) are informative because they document emboli entering the cerebral vasculature in the form of high-intensity transcranial signals (HITS). Pugsley and colleagues used this technology to demonstrate the importance of arterial line filters [1]. Other investigators have shown cerebral air associated with aortic cannula placement and perfusionist interventions [2–5], surgical events such as CPB initiation and aortic cross-clamp removal [6], and increased cerebral air with open-chamber cardiac procedures compared to closed-chamber procedures [7]. TCD also has the potential to reveal a link between cerebral emboli and poor neurologic outcomes [8].

The placement of ultrasonic bubble measuring devices at various positions on the CPB circuit during cardiac surgery enables investigators to understand the source of GME generation and explain the transmission observed in TCD studies. Investigators use these devices clinically to understand the air-trapping capabilities of various CPB disposables, such as oxygenators [9], venous bubble traps [10], and integrated versus non-integrated arterial line filters [11]. Others have used bubble counters to understand air entry associated with open vs. closed surgical procedures, perfusionist interventions, left ventricular venting, and surgical manipulation [12–14]. A benefit of measuring bubble activity in vivo is that results can be linked to neurologic testing. Doganci and colleagues demonstrated a positive correlation between intraoperative GME intensity and neurocognitive tests, suggesting that the level of GME may be a determinant of the psychological outcome after coronary artery bypass grafting with CPB [15].

The simultaneous use of ultrasonic bubble measuring devices attached to the CPB circuit and ultrasonic measurement of emboli in the cerebral arteries during surgery provides clinicians with a clearer picture of best practices in mitigating emboli during CPB. It can help develop quality improvement plans [16].

In vitro studies using ultrasonic bubble counters enable patient-independent examination of the air-handling capabilities of CPB circuit components and perfusion practices associated with increased GME. Controlled benchtop studies can minimize potential confounders associated with clinical studies and isolate the influence of variables such as air in the venous line/reservoir, air entering the cardiotomy suction ports, and the influence of the venous reservoir level.

Investigators have found that residual or entrained air in the venous cannula/line can be transmitted past the pump, oxygenator, and arterial filter [17–19]. Venous air is especially concerning when using vacuum-assisted venous drainage [20, 21]. Excessive cardiotomy suction or vent speed can also increase the number of bubbles transmitted to the arterial line. Mixing nitrogenous air with blood results in foam that can pass through the cardiotomy, pump, oxygenator, and arterial filter [22–25]. A lower reservoir level shortens the distance bubbles introduced into the venous line or cardiotomy reservoir must travel to exit and move into the arterial pump, and has been implicated in the ability of these disposables to remove air [26].

The ability to trap or remove air after it enters the cardiopulmonary bypass circuit is also vital, leading investigators to test the GME removal capability of oxygenators and arterial filters [27–30]. Recently, interest has focused on the efficacy of GME removal by arterial filters integrated into oxygenators [31–34].

The amount of GME a patient receives results from the combined impact of many variables. A recent paper elegantly described this using an in vitro circuit and ultrasonic bubble counter on the reservoir outlet to develop a neural network-based modeling of the number of microbubbles associated with four circulation factors: suction flow rate, venous reservoir level, continuous blood viscosity, and perfusion flow rate [35]. They found the field suction flow rate to be the most predictive of anticipated GME activity, while perfusion blood flow was the least predictive, as measured post-reservoir. This group also developed a five-factor model examining how temperature changes can affect GME transmission [36].

Building on the work of these investigators, this project aims to develop an understanding of how the introduction and movement of GME in the cardiopulmonary bypass circuit are related to two modifiable interventions – suction flow rate and venous reservoir level. We aimed to isolate and explore the influence of these interventions using a state-of-the-art bubble counter, testing currently used perfusion disposable reservoirs and oxygenators/arterial filters from three different manufacturers. We hypothesize that the suction flow rate will be directly proportional to GME transmission, while the reservoir level will be inversely proportional, and we aim to guide safe threshold values for each.

## Materials and methods

### Hardware

All trials were conducted using a four-pump base heart-lung machine (Stockert S3 Roller Pump with S3 Console 10-60-00, SORIN Group Deutschland GmbH, Müchen, Germany) with a Sorin Revolution centrifugal pump driver and ultrasonic flow probe (LivaNova, Mirandola, Italy) placed distal to the oxygenator. A 3T Heater/Cooler (LivaNova, Mirandola, Italy) was used to control temperature.

### Circuit description

**Figure 1:**
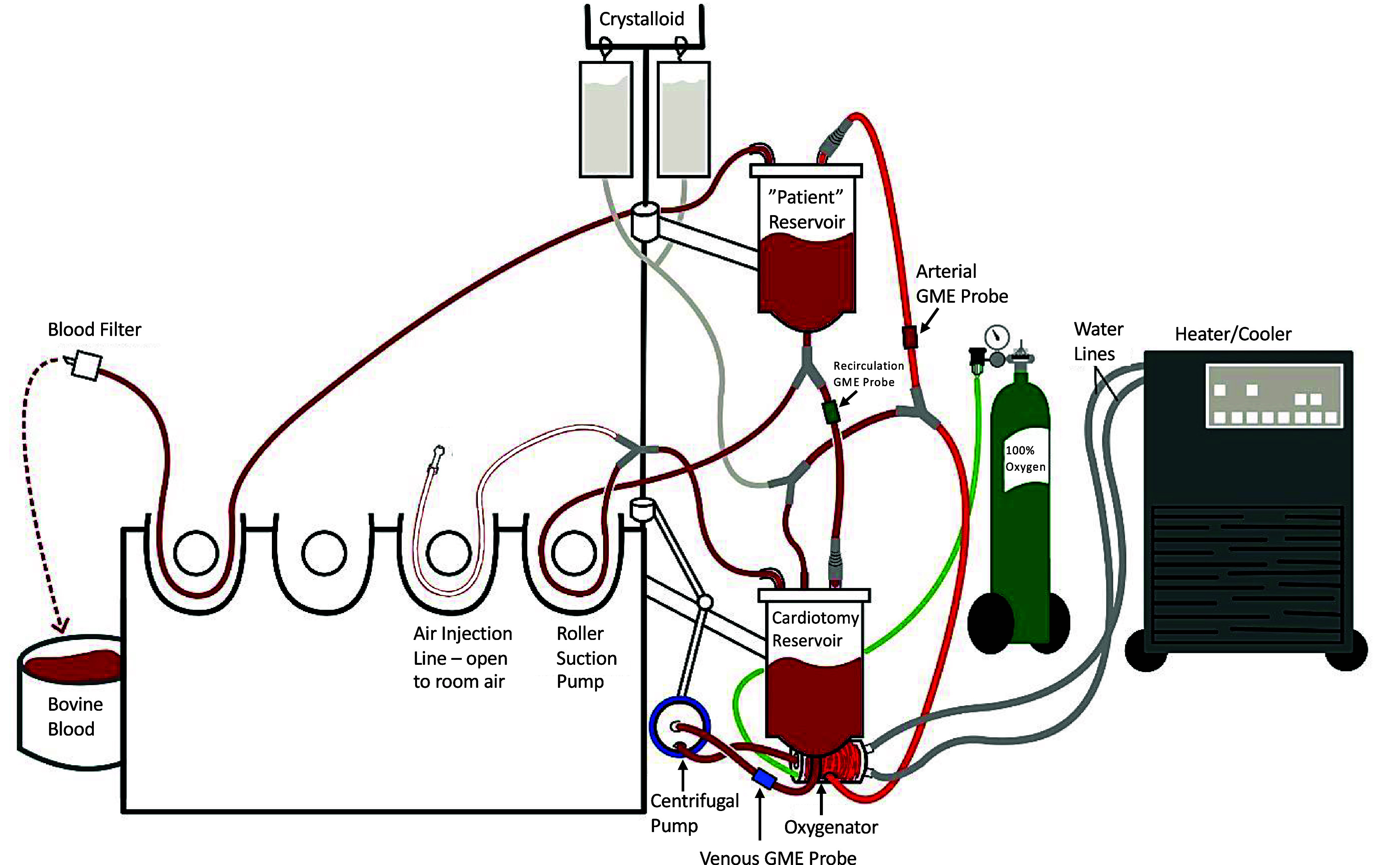
Circuit description. Schematic of the laboratory-based (in-vitro) cardiopulmonary bypass circuit. Bovine blood was recirculated from the “test” cardiotomy reservoir, past the “venous” GME sensor, to the centrifugal pump, where it was pumped through the oxygenator. Blood flowed past the “arterial” GME sensor and into the “patient” reservoir, where it drained back into the “test” reservoir after passing through the “recirculation” GME probe. Suctioned blood was pumped from the “patient” reservoir and into the suction port of the test reservoir by the roller suction pump. This suctioned blood was aerated by another roller pump that entrained room air into the suction blood flow.

The in vitro, open, recirculating test circuit (Figure 1) is a modified version of a previous design [37] used in our laboratory to mimic adult perfusion. The first modification to this circuit was adding a Terumo Capiox® FX25 Advance venous reservoir (Terumo, Ann Arbor, MI, USA) to serve as a “patient” reservoir. This functioned to minimize GME returning to the reservoir being evaluated via the venous line, allowing for independent control of level and volume in the “test” reservoir using a Hoffman clamp. We found anecdotally that it was easier to de-air the circuit before experimentation if blood was directed to this “patient” reservoir via the venous line. A second modification was adding a 1/4-inch sucker configuration to deliver a controlled mix of room air (200 mL/min) and blood into the cardiotomy portion of the “test” venous reservoir (Figure 1).

The arterial pump was a Sorin Revolution centrifugal pump (LivaNova, Mirandola, Italy) with a flow probe calibrated to zero before each trial. The roller pumps used for suction and air injection were set to 100% occlusion. PVC tubing (1/4, 3/8, ½ inch) was cut to the same standardized lengths for each experimental trial and connected with appropriate tubing connectors. Following the completion of each trial, all disposable components were discarded and replaced.

The coated “test” reservoir and oxygenator with integrated arterial filter combinations trialed were as follows: Medtronic Affinity Fusion^TM^ (*n* = 10) (Medtronic, Minneapolis, MN, USA), SORIN Inspire 8F (*n* = 9) (LivaNova, Mirandola, Italy), and Capiox® FX25 Advance (*n* = 6) (Terumo Cardiovascular, Ann Arbor, MI) (Table 1). The oxygenator manifold and arterial filter purge lines were opened during the trials, and the volume was returned to the test reservoir’s cardiotomy. Post-oxygenator line pressure was monitored and maintained at 180 mmHg by placing a partial clamp on the arterial line to simulate arterial cannulation. A filtered gas line delivered 100% tanked oxygen to the oxygenator during the trials.

**Table 1:** Specifications of membrane oxygenators and venous reservoirs tested in this study.

	Medtronic affinity fusion™	LivaNova SORIN inspire™8F	Terumo CAPIOX® FX25 advance
Oxygenator type	Microporous polypropylene hollow fiber	Microporous polypropylene hollow fiber	Microporous polypropylene hollow fiber
Oxygenator filtration	25 μm progressive depth	38 μm screen	32 μm screen
Oxygenator maximum blood flow rate	7.0 L/min	8.0 L/min	7.0 L/min
Reservoir type (Hardshell)	Dual chamber	Dual chamber	Dual chamber
Reservoir capacity	4,500 mL	4,500 mL	4,000 mL
Reservoir minimum operating level	200 mL @ 7 L/min	150 mL	150 mL
Reservoir maximum blood flow rate	7.0 L/min	8.0 L/min	7.0 L/min
Cardiotomy reservoir filtration	30 μm	41 μm	47 μm
Oxygenator /reservoir coating	Balance™	PHISIO™	Xcoating™

Medtronic information – accessed 7/31/2024, LivaNova information – accessed 7/30/2024, Terumo information – accessed 7/30/2024.

### Priming procedure

The circuit was flushed with 4 L of carbon dioxide for 5 min before priming. Next, it was primed with 2 L of crystalloid (Normosol-R). It circulated for 10 min through a 0.2-micron pre-bypass filter (Pall Corporation®, Portsmouth, PA) to remove particulates and residual air.

Five liters of fresh bovine blood were collected for each trial from a local slaughterhouse and mixed with 30,000 IU of heparin to prevent clotting during transportation. Upon arrival at our facility, we measured the activated clotting time (Hemochron Signature Elite, Werfen Bedford, MA, USA) and the spun hematocrit (Model CMH30, UNICO, Dayton, NJ, USA).

After removing air and the pre-bypass filter from the circuit, a calculated volume of filtered and anticoagulated bovine blood was introduced to the test circuit while simultaneously displacing the same crystalloid volume to achieve a goal hematocrit of 25%. Additional heparin was administered to maintain an activated clotting time of over 480 s. Trials were started within one to two hours of blood collection and completed within four to five hours after blood collection.

### GME measurement

A pulsed ultrasonic Doppler system, the Bubble Counter Clinical BCC300 (Gampt, Zappendorf, Germany), was used to measure the air generated during each trial. This device counts the number of bubbles passing through the probes each second, measures the diameter, and derives the bubble volume. Bubbles were measured at three locations using 3/8-inch clamp-on probes coated in ultrasonic gel (Figure 1). The first sensor was placed three inches below the exit of the tested reservoir, between the reservoir and the centrifugal pump, and designated as the “venous” sensor. The second sensor was placed on the arterial outflow (post-oxygenator/filter), twelve inches before the venous inlet on the patient reservoir, and designated as the “arterial” sensor. The final sensor was placed three inches below the exit of the patient reservoir before the venous inlet of the test reservoir and was designated as the “recirculation” sensor. Trial data collection and interventions did not begin until all probes were clear of GME.

After each trial, raw data were exported from the BCC300 onto a thumb drive as an MS Excel file. Markers were placed with each intervention, which was also displayed on the spreadsheet. The first three minutes (180 s) of bubble count data were then formatted and loaded onto a PC for analysis.

### Testing conditions

To mitigate confounding variables, testing conditions during all experimental trials were as follows: Arterial blood flow was recirculated at 4.5 L/min, and blood temperature measured at the oxygenator outlet was maintained at 37° C. 100% oxygen was administered into the oxygenator gas in port at 4.5 L/min sweep to match blood flow. The hematocrit was maintained between 23% and 25%, and the activated clotting time was maintained above 480 s following heparin administration, which was acceptable to begin the trial [38]. Air administration into the pump sucker tubing was continuous at 0.20 L/min except during baseline measurements when it was turned off. The variables altered and assessed during the experiment were the test reservoir level and the suction roller pump suction speed.

### Protocol

**Figure 2:**
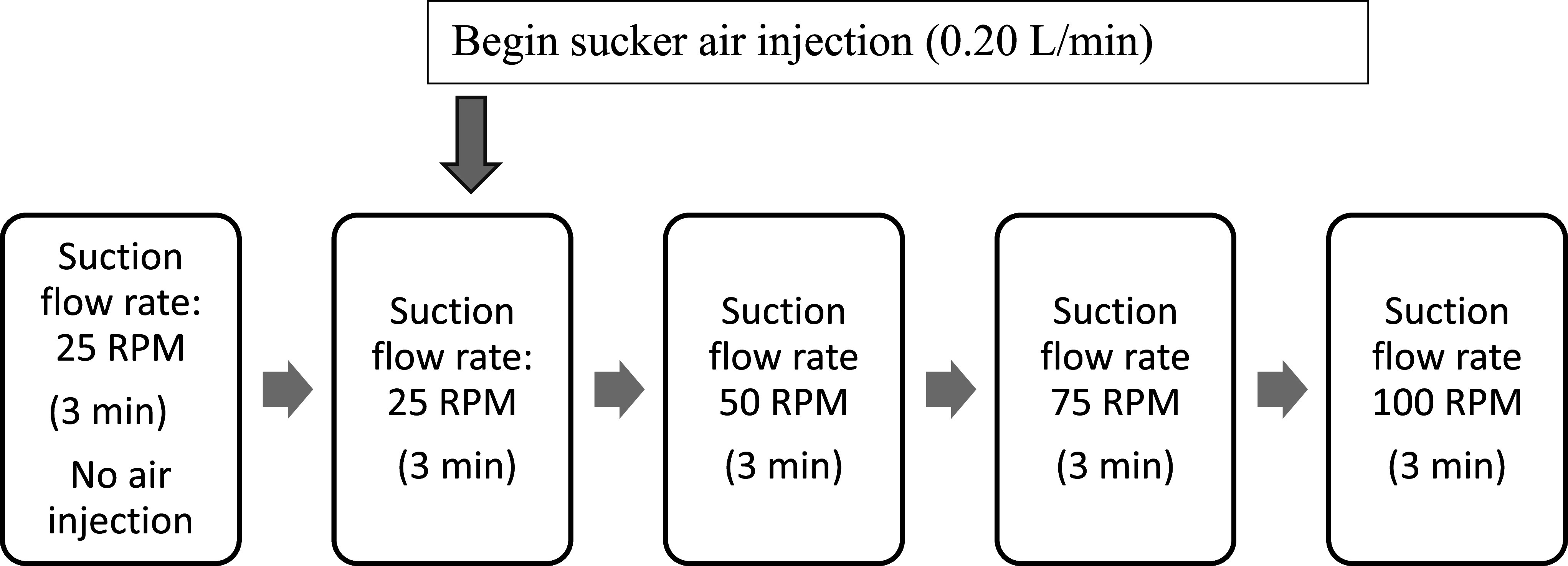
Data collection protocol. The sequence of data collection at each reservoir level (200 mL, 500 mL, 1000 mL) was identical. 1. Arterial centrifugal pump flow was set at 4.5 L/min, gas flow 4.5 L/min with 100% oxygen and arterial temperature was 37C. 2. Suction flow rate was set at 25 RPM (0.32 L/min) initially with no sucker air injection for a “no air” verification recording. 3. Sucker air injection was changed to 0.2 L/min, then data was recorded for suction flow rates of 25 RPM (0.32 L/min) with air, 50 RPM (0.65 L/min) with air, 75 RPM (0.99 L/min) with air, and 100 RPM (1.32 L/min) with air. 4. Sucker air injection was stopped, and this sequence was repeated at levels of 500 mL and 1000 mL. Data was collected from the Gampt BCC300 continuously throughout the protocol, and markers were applied to denote interventional changes and start/stop times. The first three minutes (180 s) of bubble data were analyzed for each intervention.

After thoroughly de-airing the blood-primed circuit, the arterial centrifugal pump flow was set at 4.5 L/min, and a 100% oxygen sweep was matched to the blood flow. The arterial temperature was maintained at 37 °C. The sequence of interventions and data collection (Figure 2) was identical at each “test reservoir” level (200 mL, 500 mL, and 1000 mL). The level was set at the desired value by manipulating a Hoffman clamp on the “patient reservoir” outlet line. During each intervention, bubble count data from three probes (venous, arterial, and recirculation) were measured simultaneously.

**Figure 3:**
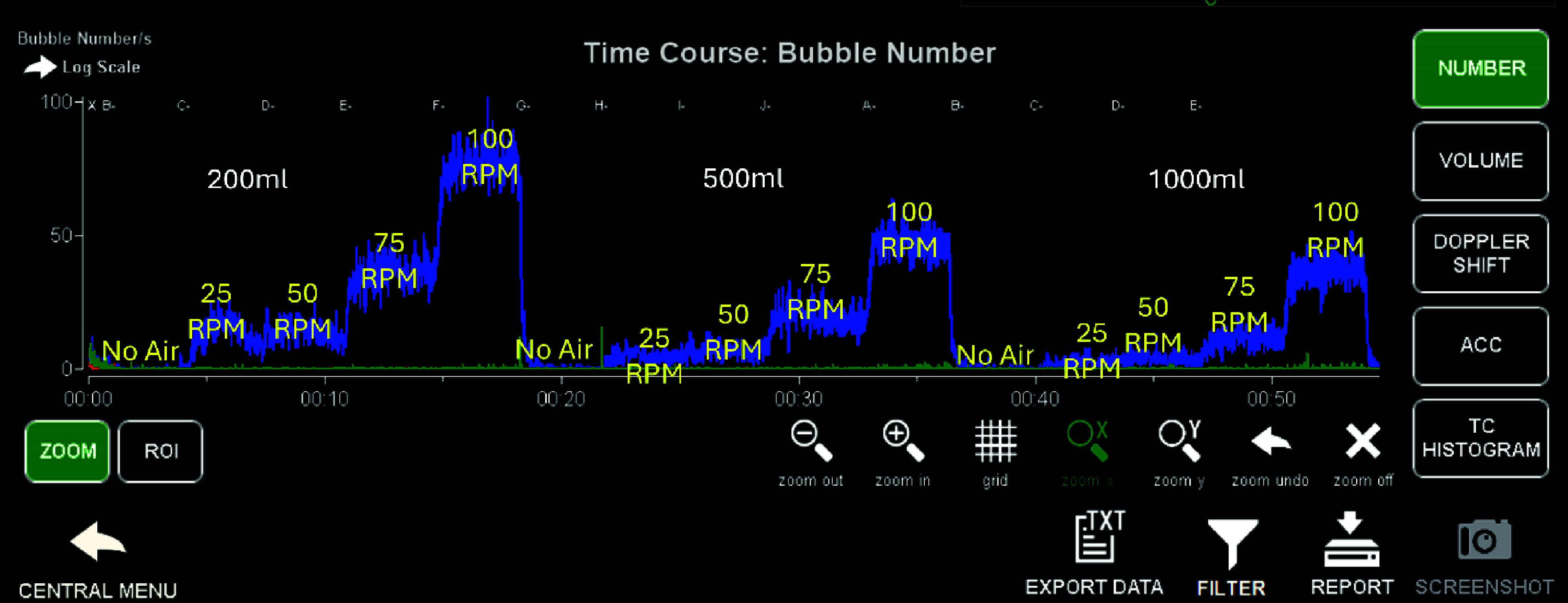
GAMPT bubble count display. Gampt BCC300 bubble number display screen with a logarithmic scale showing a real-time display of bubble number (count) with intervention changes. Overlayed reservoir level (200 mL, 500 mL, 1000 mL) is shown left to right with the corresponding sucker speeds (25 RPM with no air, 25 RPM with air, 50 RPM with air, 75 RPM with air, and 100 RPM with air).

Baseline measurements were made at a sucker speed of 25 RPM (0.32 L/min) with no air injection into the suckers. The air injection roller pump was then started and increased to 0.20 L/min to introduce room air into the sucker blood returning to the test reservoir. Measurements with aerated sucker return were then obtained at 25 RPM (0.32 L/min), 50 RPM (0.65 L/min), 75 RPM (0.99 L/min), and 100 RPM (1.32 L/min). The air injection pump was then stopped, the level reset to the desired value, and this sequence was repeated. The bubble count per second was measured continuously by the Gampt BCC300 throughout the protocol, and markers were applied to denote interventional changes and start/stop times. The first three minutes (180 s) of bubble data were summed for each intervention. Figure 3 shows the Gampt BCC300 bubble number display screen, which features a logarithmic scale, providing a real-time display of the bubble number (count) with changes resulting from interventions.

Twenty-five trials were completed at a reservoir level of 200 mL, and 23 at reservoir levels of 500 mL and 1000 mL. Of these trials, ten were completed using Medtronic Affinity Fusion, and six were completed using Terumo CAPIOX FX25 Advance at each reservoir level (200 mL, 500 mL, and 1000 mL). The LivaNova SORIN Inspire 8F reservoir underwent nine trials at 200 mL, 7 trials at 500 mL, and seven trials at 1000 mL.

### Statistical analysis

Three minutes (180 s) of bubble count was recorded for each of the three sensors and each intervention, and the results were averaged for all trials. The statistical software used to analyze the collected data was “R” [39], which is used for statistical computing and graphics. Box plots were made to visualize the group differences in mean and variability. A two-way ANOVA was utilized to test differences in the average total number of GME (dependent variable) with two independent variables: suction flow rate and reservoir level. A post-hoc Tukey Honest Significant Difference (HSD) test was performed to determine whether the mean difference between specific pairs of groups was statistically significant. The alpha level for statistical significance is 0.05.

## Results

### Venous GME count

**Figure 4:**
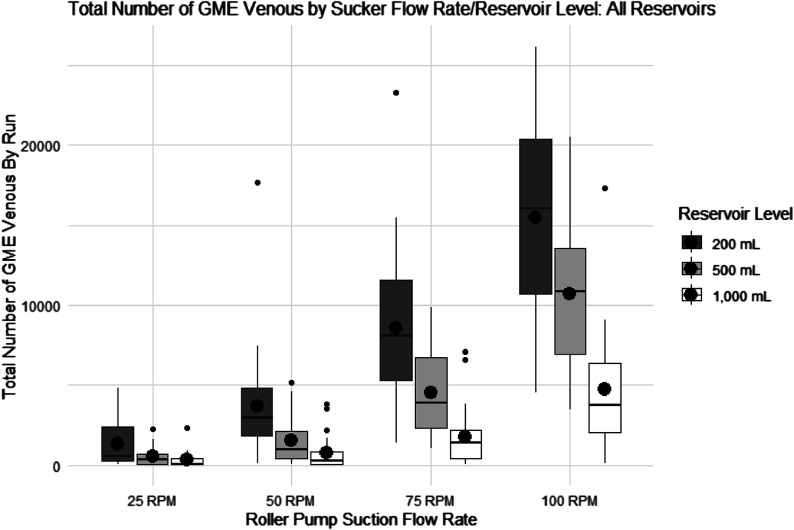
Box plot of post-reservoir (venous) GME count data. Box plot showing the total number of GME measured for 3 min (180 s) by the “venous” sensor for suction flow rate and reservoir levels, including all reservoir types (Medtronic Affinity Fusion, Liva Nova SORIN Inspire 8F, and Terumo CAPIOX FX25 Advance). Roller pump suction flow rate: 25 RPM (0.32 L/min), 50 RPM (0.65 L/min), 75 RPM (0.99 L/min), and 100 RPM (1.32 L/min), and reservoir level (mL) is on the *x*-axis, and total bubble number is on the *y*-axis. The lines in each boxplot represent the median values, and the black circles in each boxplot represent the averages.

Summary statistics describing the total number of GME measured over 3 min (180 s) by the “venous” sensor are shown (Figure 4), along with a box plot illustrating data from all reservoirs tested (Medtronic Affinity Fusion, LivaNova SORIN Inspire 8F, and Terumo CAPIOX FX25) combined. Box plots were made to visualize the group differences in Mean and variability. The independent variables, four suction flow rates (25 RPM (0.32 L/min), 50 RPM (0.65 L/min), 75 RPM (0.99 L/min), and 100 RPM (1.32 L/min) and three reservoir levels (200, 500, 1,000) mL are shown on the *x*-axis with GME number on the *y*-axis. The results of the Multivariate Shapiro-Wilk normality test indicate that the data are not normal (*p* < 0.0001). However, given the sample size of 284 observations in the data, the detection of minor discrepancies in non-normality is expected.

The results of the two-way ANOVA examining differences in the average total number of GME for the venous sensor, by roller pump suction flow rate and reservoir level, indicate a statistically significant interaction between roller pump suction flow rate and reservoir level (*p*-value < 0.0001). This tells us that the combined effect (interaction) of the suction flow rate and the reservoir level on the GME number is statistically significant. GME number increased with increased sucker flow rate and decreased with increased reservoir level (Figure 4).

Table 2 shows post-hoc comparisons of the average difference in GME number by roller pump suction speed at constant venous reservoir levels. Statistically significant differences are denoted by *p* < .05 and are shown in bold, with adjustments made using the Tukey HSD method. The effect of suction speed was most pronounced at the lowest reservoir level (200 mL). GME number increased significantly as sucker speed increased, except between 25 and 50 RPM. As the level increased to 500 mL, the GME number significantly increased as sucker speed increased, except between 25–50 RPM and 50–75 RPM. At the highest level tested (1000 mL), the effect of suction speed was least evident. The only significant difference in GME number was detected between 25–100 RPM and 50–100 RPM.

**Table 2:** Statistical analysis for the “venous” GME sensor for all test reservoirs combined. This shows post-hoc comparisons of the average difference in GME number by roller pump suction speed: 25 RPM (0.32 L/min), 50 RPM (0.65 L/min), 75 RPM (0.99 L/min), and 100 RPM (1.32 L/min) at a constant venous reservoir level (200 mL, 500 mL, 1000 mL). Statistically significant differences are denoted by *p* < .05 and are shown in bold, with adjustments made using the Tukey HSD method.

Reservoir level	Suction speed 1	Suction speed 2	Difference in average GME number	Adjusted *P*-value
200 mL	25 RPM	50 RPM	2284.12	0.371
200 mL	**25 RPM**	**75 RPM**	**7239.84**	**0**
200 mL	**25 RPM**	**100 RPM**	**14112.36**	**0**
200 mL	**50 RPM**	**75 RPM**	**4955.72**	**0**
200 mL	**50 RPM**	**100 RPM**	**11828.24**	**0**
200 mL	**75 RPM**	**100 RPM**	**6872.52**	**0**
500 mL	25 RPM	50 RPM	981.391	0.997
500 mL	**25 RPM**	**75 RPM**	**3997.652**	**0.003**
500 mL	**25 RPM**	**100 RPM**	**10142.609**	**0**
500 mL	50 RPM	75 RPM	3016.261	0.084
500 mL	**50 RPM**	**100 RPM**	**9161.217**	**0**
500 mL	**75 RPM**	**100 RPM**	**6144.957**	**0**
1,000 mL	25 RPM	50 RPM	470.565	1
1,000 mL	25 RPM	75 RPM	1467	0.936
1,000 mL	**25 RPM**	**100 RPM**	**4397.087**	**0.001**
1,000 mL	50 RPM	75 RPM	996.435	0.997
1,000 mL	**50 RPM**	**100 RPM**	**3926.522**	**0.004**
1,000 mL	75 RPM	100 RPM	2930.087	0.107

Table 3 presents post hoc comparisons of the average difference in GME number by venous reservoir level at a constant roller pump suction speed to examine the venous data from a different perspective. Statistically significant differences are denoted by *p* < .05 and are shown in bold, with adjustments made using the Tukey HSD method. There were no significant differences in bubble numbers at suction speeds of 25 RPM and 50 RPM as the level increased from 200 mL to 1000 mL. At a suction speed of 75 RPM, the average difference in GME number exhibited an indirect relationship with level and showed significant changes at reservoir levels of 200–500 mL and 200–1000 mL. At a suction speed of 100 RPM, all changes in level produced significant differences in bubble numbers.

**Table 3:** Statistical analysis for the “venous” GME sensor for all test reservoirs combined. This shows post-hoc comparisons of the average difference in GME number by reservoir level (200 mL, 500 mL, 1,000 mL) at a constant suction speed of 25 RPM (0.32 L/min), 50 RPM (0.65 L/min), 75 RPM (0.99 L/min), and 100 RPM (1.32 L/min). Statistically significant differences are denoted by *p* < .05 and are shown in bold, with adjustments made using the Tukey HSD method.

Suction speed	Reservoir level 1	Reservoir level 2	Difference in average GME number	Adjusted *P*-value
25 RPM	200 mL	500 mL	814.793	0.999
25 RPM	500 mL	1000 mL	234.391	1
25 RPM	200 mL	1000 mL	1049.184	0.994
50 RPM	200 mL	500 mL	2117.522	0.528
50 RPM	500 mL	1000 mL	745.217	1
50 RPM	200 mL	1000 mL	2862.739	0.109
**75 RPM**	**200 mL**	**500 mL**	**4056.981**	**0.002**
75 RPM	500 mL	1000 mL	2765.044	0.164
**75 RPM**	**200 mL**	**1000 mL**	**6822.024**	**0**
**100 RPM**	**200 mL**	**500 mL**	**4784.544**	**0**
**100 RPM**	**500 mL**	**1000 mL**	**5979.913**	**0**
**100 RPM**	**200 mL**	**1000 mL**	**10764.457**	**0**

### Arterial GME count

**Figure 5:**
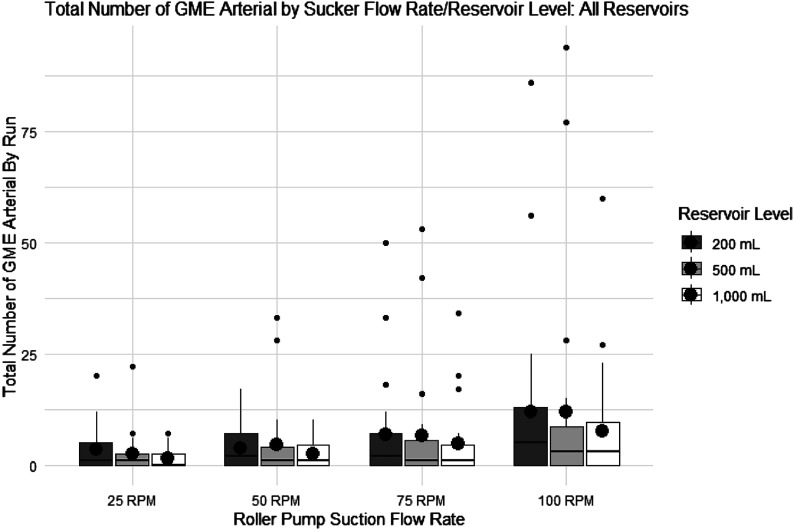
Box plot of post-oxygenator/filter (Arterial) GME count data. Box plot showing the total number of GME measured for 3 min (180 s) by the “arterial” sensor for suction flow rate and reservoir levels, including all reservoir types (Medtronic Affinity Fusion, Liva Nova SORIN Inspire 8F, and Terumo CAPIOX FX25 Advance). Roller pump suction flow rate: 25 RPM (0.32 L/min), 50 RPM (0.65 L/min), 75 RPM (0.99 L/min), and 100 RPM (1.32 L/min), and reservoir level (mL) is on the *x*-axis, and total bubble number is on the *y*-axis. The lines in each boxplot represent the median values, and the black circles represent the averages.

Summary statistics describing the total number of GME measured over 3 min (180 s) by the “arterial” sensor are presented (Figure 5), accompanied by a box plot illustrating data from all reservoirs tested (Medtronic Affinity Fusion, LivaNova SORIN Inspire 8F, and Terumo CAPIOX FX25) combined. Box plots were made to visualize the group differences in mean and variability. The independent variables, four suction flow rates (25 RPM (0.32 L/min), 50 RPM (0.65 L/min), 75 RPM (0.99 L/min), and 100 RPM (1.32 L/min) and three reservoir levels (200, 500, 1000) mL are shown on the *x*-axis with GME number on the *y*-axis. Note the scaling difference between Figures 4 and 5: the amount of GME flowing past the arterial sensor (post-oxygenator/arterial filter) was significantly less than that found with the venous sensor (post-reservoir).

As with the venous GME number, this data was not statistically normal; however, given the large sample size of 284 observations, a two-way ANOVA was used to statistically evaluate GME number differences between suction flow rates and reservoir levels. Unlike the venous GME sensor, the data from the arterial GME sensor revealed no statistical interaction between suction flow rate and reservoir level (*p*-value = 0.9946) or between reservoir level and GME count (*p*-value = 0.2919). Changes in suction flow rate overall (25–100 RPM) did, however, affect the GME number from the arterial sensor (*p*-value = 0.0005).

Table 4 shows the post hoc comparisons using Tukey HSD to compare suction flow rates. The adjusted *p*-values in bold are statistically significant, with an adjusted *p*-value of less than 0.05. Significant differences in average bubble number were only noticed between 25–100 RPM and 50–100 RPM.

**Table 4:** Statistical analysis for the “arterial” GME sensor for all test reservoirs combined. This shows post-hoc comparisons of the average difference in GME number by suction speed 25 RPM (0.32 L/min), 50 RPM (0.65 L/min), 75 RPM (0.99 L/min), and 100 RPM (1.32 L/min). Statistically significant differences are p < .05, shown in bold, and adjusted using the Tukey HSD method.

Roller pump suction flow rate comparisons	Difference in averages	Adjusted *P*-value
50 RPM-25 RPM	1.084507	0.9494825
75 RPM-25 RPM	3.492958	0.3074894
**100 RPM-25 RPM**	**7.943662**	**0.0005831**
75 RPM-50 RPM	2.408451	0.6296268
**100 RPM-50 RPM**	**6.859155**	**0.0041821**
100 RPM-75 RPM	4.450704	0.1228292

### GME size analysis

**Figure 6:**
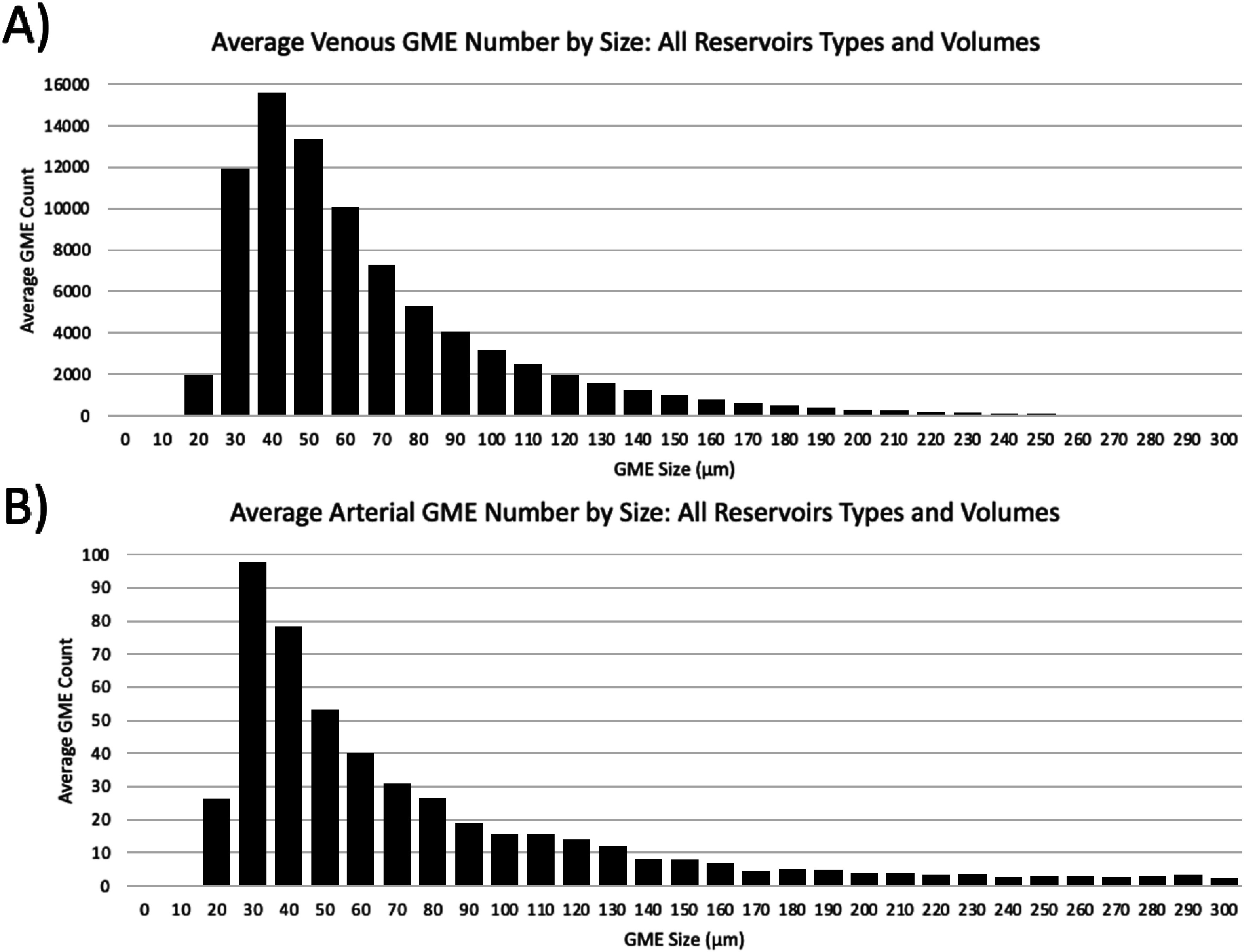
Average GME count and size data. Average (A) venous and (B) arterial GME count and size for all 25 trials, reservoir types (Medtronic Affinity Fusion, LivaNova SORIN Inspire 8F, and Terumo CAPIOX FX25), levels (200 mL, 500 mL, and 1000 mL) and suction flow rates (25 RPM (0.32 L/min), 50 RPM (0.65 L/min), 75 RPM (0.99 L/min) and 100 RPM (1.32 L/min)).

Venous and arterial GME size and amount were recorded for all reservoir types (Medtronic Affinity Fusion, LivaNova SORIN Inspire 8F, and Terumo CAPIOX FX25), levels (200 mL, 500 mL, and 1,000 mL) and suction flow rates (25 RPM (0.32 L/min), 50 RPM (0.65 L/min), 75 RPM (0.99 L/min) and 100 RPM (1.32 L/min)) for all trials. The total venous (Figure 6A) and arterial (Figure 6B) GME data were categorized by size, ranging from 0 to 300 μm, and averaged across all 25 trials.

## Discussion

### Summary of findings

One of this project’s goals was to develop an in vitro model that is both explanatory and predictive of GME presence and transmission in a cardiopulmonary bypass circuit. After completing 25 trials and examining the resultant data, the model described in this study helped explain how varying “circulation factors,” as designated by Miyamoto et al. [36], can influence a measurable consequence (GME count). This study focused on the influence of two factors; however, the developed model can be adapted to many experimental protocols.

Another goal of this study was to replicate previous investigations examining the relationship between two modifiable interventions – suction speed and reservoir level – and their influence on GME count measured in both venous (post-reservoir) and arterial (post-oxygenator) lines. We selected disposable components from three manufacturers commonly used in the United States today and the state-of-the-art bubble counter (Gampt BCC300) to enhance the applicability to current perfusion practice.

When keeping other “circulation factors” constant, we found that both reservoir level and suction speed influenced GME count, as measured by the venous (post-reservoir) and arterial (post-oxygenator) sensors. Notably, we found a statistical interaction between these variables at the venous sensor, indicating that suction speed and level interact combinatorially. The effect of suction speed influenced the impact of the level and vice versa. This suggests, in a general sense, that as the reservoir level lowers, it would be prudent to monitor closely and, if possible, minimize suction speed to prevent the transmission of GME. This interaction effect was not evident with measurements from the arterial sensor.

The suction/vent flow rate should be continuously monitored and adjusted as needed during CPB. It must be high enough to prevent the surgical field from being flooded with blood, but low enough to minimize the admixture of air with blood. The reservoir level during CPB is influenced by several variables, including the patient’s blood volume, circuit prime volume, and venous return. A minimum operating reservoir level is designated by the reservoir manufacturer (Table 1) and depends on the arterial flow rate and the desired reaction time to emptying.

This study demonstrated that more bubbles were detected at the venous (post-reservoir) sensor as suction speed increased. The integrated cardiotomy reservoir was unable to capture all the aerated blood from the sucker, and some of it passed out of the reservoir and into the centrifugal pump. At all three tested levels (200 mL, 500 mL, and 1,000 mL), no statistically significant change in post-cardiotomy bubble count was detected when suction speed was maintained between 25 and 50 RPMs. This corresponds with a flow rate of less than 0.65 L/min (Table 2). Increasing the suction flow to 75 RPMs (approximately 1.0 L/min) significantly changed the bubble count at 200 mL and 500 mL levels. At the maximum suction speed evaluated (100 RPM or 1.32 L/min), significant changes in bubble count were found at all three levels. Interestingly, even with 1 L of blood in the reservoir, bubbles bypassed the venous sensor at the maximum suction flow.

Table 4 displays the results from the arterial sensor (post-oxygenator/filter). As expected, most bubbles measured as they exited the reservoir were either trapped by the integrated arterial filter or diffused across the hollow fiber membrane. Hence, the differences in the average bubble counts between changes in sucker flow were much less compared with those from the venous sensor. Significant changes in bubble count were observed as the suction speed increased from 25 to 100 RPM and from 50 to 100 RPM, indicating that at these higher suction flows, bubbles can still escape from the oxygenator and enter the patient. No significant interaction was found between level and suction speed using the arterial sensor data, as observed with the venous sensor. Additionally, the reservoir level did not appear to influence the amount of arterial air detected.

The size of the GME, detected in micrometers, was averaged across all our runs and is displayed in Figure 6. The venous (post-reservoir) sensor captured a wide range of bubble sizes, with most falling between 30 and 80 microns, with some significant outliers. The arterial sensor’s distribution appeared similar, but it was interesting that many bubbles, less than 40 microns in diameter, were observed. The arterial filter pore size of the oxygenators tested ranged from 25 to 38 microns, which may explain why many bubbles detected were less than 40 microns.

### Mechanism

This study did not directly investigate the mechanism causing the observed increase in bubble transmission with increased suction speed and changes in reservoir level. Interfacial bubbles (bubbles on a liquid) tend to fractionate, creating numerous smaller “daughter” bubbles rather than disappearing [40]. In this study, fractionation or bubble rupture might depend on how quickly the aerated blood shuttles into the reservoir (suction speed), causing bubble rupture. Once these larger bubbles rupture, the distance they travel to the reservoir exit (reservoir level) plays a role. The mechanism describing their movement is multifactorial and can relate to blood viscosity, blood components that can carry the micro-air through the circuit, and the design of the reservoir, particularly at air-blood mixing surfaces.

Blood viscosity may affect this transmission. Mitchell et al. demonstrated that blood viscosity is directly proportional to GME generation and transmission [26]. We used bovine blood hematocrit as a proxy for viscosity, which was kept constant in this study, but we did not directly measure blood viscosity. In clinical settings, viscosity continuously fluctuates due to blood transfusions or fluids administered during surgery [35]. While these changes are typically minor, they may still affect the amount of GME transmitted.

In addition to blood viscosity, blood composition plays a role in GME transmission. Preliminary trials conducted before the commencement of our work revealed that circuits containing crystalloids yielded minimal GME creation and transmission. Using glycerin, matched to a blood hematocrit of 25%, showed more GME transmission when compared with crystalloid, but still less compared to bovine blood. These findings align with similar work by Harea et al., supporting the hypothesis that blood proteins may facilitate GME transmission [41].

Another potential mechanism contributing to GME transmission occurs at the reservoir’s air-blood interface. The reservoir design may explain discrepancies in GME count when comparing manufacturer reservoirs. It speaks to the reservoir’s ability to promote bubble buoyancy, keep big bubbles big, and remove or filter the air. At this interface, inadvertent mixing of blood and air creates a foam that ruptures, generating numerous smaller bubbles into the circuit [40]. CPB circuits are designed to remove macro-air effectively but are less efficient with micro-air. The air-blood interface remains an unavoidable factor when utilizing open venous reservoirs. In the future, we hope to compare manufacturer reservoirs and explore how reservoir design might play a role in GME transmission.

While there are many potential mechanisms behind GME transmission, improving procedural practices can help reduce the amount of GME transmitted to the patient. By refining techniques to minimize these contributing factors, it may be possible to reduce GME exposure during CPB and improve patient outcomes.

### Clinical implications

Introducing large air bubbles into the arterial circulation is a well-established iatrogenic cause of cerebral ischemia, which can result in multifocal ischemic injury or even death [42, 43]. While various CPB techniques and equipment, such as level sensors and gross air detectors, are employed to prevent the transmission of large air bubbles, small, unfiltered air bubbles may still pose a risk. The clinical question that needs to be answered is whether GME harms the patient. Unfortunately, the literature on this issue is divided, and the GME load required to produce neurologic impairment in humans is difficult to establish. Current research is focused on determining how GME load can affect potential occlusion of critical capillary beds, endothelial irritation, and the inflammatory response.

Managing iatrogenic risks of GME introduction remains challenging, and the process may be more patient-dependent and multifactorial than previously thought [44]. GME, fat, and other microemboli can deform within the brain, disrupting the blood-brain barrier and contributing to neurological injury [45]. Other organs are also impacted by the systemic effects of microemboli [14, 45–47]. These smaller bubbles can directly block capillaries or coalesce into larger bubbles, thereby increasing the risk of vascular obstruction. Most in vivo studies investigating the pathological effects of air bubbles introduce known volumes of air; however, the size (diameter) of the bubbles is often not specified, which is crucial when examining GME [48]. Moreover, air can be introduced into the venous circulation, compromising pulmonary function, or paradoxically enter the arterial circulation through atrial septal defects [43].

Aside from stroke risk, postoperative cognitive dysfunction (POCD) remains a significant concern. As with air introduction, POCD is multifactorial and patient dependent. Contributing factors include the number, volume, and type (lipid or air) of emboli [43, 49]. These complex and varied causes make it challenging to establish a clear threshold of GME that leads to neurological impairment [43, 45].

Growing evidence suggests that neurological complications following CPB are not always due to significant ischemic events but instead to endothelial irritation and inflammation caused by microemboli [45, 46]. GME is a potent endothelial irritant that triggers an inflammatory cascade. When a GME contacts the endothelial surface, an inflammatory response is prompted since it is perceived as a foreign insult. As CPB progresses, inflammatory markers accumulate throughout the body, contributing to a systemic inflammatory response associated with increased morbidity and mortality. However, some studies have failed to demonstrate a significant relationship between GME and inflammatory biomarkers [50]. In addition to increased GME, another problem related to using a cardiotomy suction reservoir is the blood suctioned from the surgical field. This blood contains vasoactive cytokines and inflammatory markers, which are not filtered before being reintroduced into the patient. Once reintroduced, these substances exacerbate endothelial inflammation, contributing to further complications [9, 51–53].

Whether or not the evidence suggests that tiny bubbles may cause harm to a patient, it seems logical to take practical steps to minimize perioperative GME exposure. Our study found that GME load can be reduced by implementing easily implemented protocols, such as maintaining safe suction speeds and reservoir levels.

## Limitations of the study

It is noteworthy that this study measured GME for only 3 min. In preparation for this study, we performed several test runs lasting over an hour with continuous aerated pump suction. We found that the same level of GME activity was maintained throughout the entire period. A typical CPB case may last significantly longer, suggesting that the amount of GME passing through the circuit and then to the patient could be substantial. We also only utilized one sucker. Perfusionists often have multiple suction and vent pumps running simultaneously, which would also increase the GME count beyond our findings. The amount of air injected into the sucker flow in this study was 0.20 L/min for all the runs. It would be difficult to determine how much air gets entrained from pump suction during a clinical case, but the rate we chose was similar to that seen on CPB. Although our results indicate that the blood suction flow rate affects GME transmission, it would be interesting to see if varying the airflow would increase GME count or if GME depended more on the suction blood flow rate.

Due to the study design, we were unable to compare the differences between reservoir and oxygenator manufacturers. To compare different reservoirs and oxygenators accurately, we should have tested all three manufacturers’ circuits using the same biological replicate (a pail of bovine blood). Therefore, we pooled the results of all reservoir and oxygenator manufacturers.

This study also cannot account for the complexity of clinical practices, where manual and procedural actions can introduce additional GME into the circuit. Perfusionists often operate both suckers and vents simultaneously, and the surgeon’s manipulation of the venous and arterial lines can significantly increase GME levels. Due to these factors, the GME produced in this controlled study is likely an underestimation compared to what occurs in clinical practice. Another difference from clinical practice is that suctioned blood typically comes from the chest cavity and contains fat and other particulates. The blood suctioned in this study was from the patient reservoir and did not contain these particles.

The study used bovine blood, which can introduce variations in viscosity, coagulation factors, and platelet composition compared to human blood. We diluted the bovine blood to achieve hematocrit and viscosity levels comparable to those found during CPB in humans. Previous studies have demonstrated that bovine blood can serve as an effective substitute for human blood in GME investigations, without significantly affecting the measurement or formation of GME [38]. If bovine blood did influence GME formation, it would likely behave similarly to human blood, as bovine blood is a reliable substitute in dynamic in vitro studies. Despite these precedents, the generalizability of the findings to human physiology remains limited.

Another consideration pertains to the measurement of GME. The two most widely used GME counters, the EDAC quantifier and the Gampt BCC, have been employed in clinical and in vitro settings. However, neither is without potential error. Studies have shown that both devices may underestimate GME count at higher flow rates and overestimate it at lower flow rates [54]. Segers et al. found that the Gampt BCC tends to overestimate GME size, while the EDAC quantifier tends to overestimate GME concentration [55]. Additionally, there has been concern about whether these counters can reliably distinguish between particulate matter and true GME [7]. Despite these limitations, existing studies support the overall accuracy and clinical utility of GME counters [56].

Further limitations include the generalizability of the findings and their direct correlation to patient outcomes. Although the study used equipment and procedures commonly found in the operating room (OR), some differences in the experimental setup may have influenced GME counts. While the lab conditions were designed to replicate OR practices, the controlled lab environment may not fully replicate the dynamic and variable conditions encountered in actual clinical settings. In the OR, factors such as suction and venting are continuously adjusted, which can affect flow rates and GME transmission. Moreover, there are currently no standardized criteria for defining GME size, volume, or duration of exposure that correlate with adverse patient outcomes. As such, any attempt to infer such correlations to neurocognitive outcomes should be approached with caution [54].

Nevertheless, despite concerns regarding generalizability and direct correlation to patient outcomes, there is widespread agreement that minimizing GME within the CPB circuit is beneficial. Therefore, measures to reduce the introduction of GME should be prioritized to improve patient safety and outcomes.

## Conclusion

This study provides a unique investigation into the measurement and identification of gas microemboli (GME) transmission. While GME counters have been used in both in vitro studies and clinical practice, their application for research in clinical settings remains limited due to confounds. The in vitro design of this study offers the advantage of isolating key variables, such as air introduction, pump speed, and reservoir level, enabling a controlled assessment of their effects on GME. This feature is not possible in clinical practice. Additionally, the study employs the latest version of the state-of-the-art bubble counter, the Gampt BCC300, which enhances accuracy and data collection capabilities. By evaluating commonly used reservoirs and oxygenators currently used in clinical practice, this study helps represent what occurs during CPB procedures today.

We recommend, based on this data, that the suction speed going to a cardiotomy (single or multiple suckers) be kept to a minimum effective flow rate for any reservoir level, given their interactive combined effect on GME transmission. A worst-case and hopefully infrequent scenario, sucker bypass, where suction is high and the level is low, may introduce many GME to the patient. In general, however, suction speed and level should be continuously monitored during CPB and adjusted to prevent excessive GME transmission. Based on our results, it appears reasonable to maintain a suction speed of 50 RPM or lower (0.65 L/min) to minimize GME transmission, particularly when the reservoir level is less than 500 mL.

## Data Availability

The data are available from the corresponding author upon request.
